# Enhancement of Biomass and Lipid Productivities of Water Surface-Floating Microalgae by Chemical Mutagenesis

**DOI:** 10.3390/md15060151

**Published:** 2017-05-27

**Authors:** Daisuke Nojima, Yuki Ishizuka, Masaki Muto, Asuka Ujiro, Fumito Kodama, Tomoko Yoshino, Yoshiaki Maeda, Tadashi Matsunaga, Tsuyoshi Tanaka

**Affiliations:** Division of Biotechnology and Life Science, Institute of Engineering, Tokyo University of Agriculture and Technology, 2-24-16, Naka-cho, Koganei, Tokyo 184-8588, Japan; d-nojima@cc.tuat.ac.jp (D.N.); s168528s@st.go.tuat.ac.jp (Y.I.); muto.masaki62@chugai-pharm.co.jp (M.M.); alsouvkeas_you@yahoo.co.jp (A.U.); rc784872@bc5.so-net.ne.jp (F.K.); y-tomoko@cc.tuat.ac.jp (T.Y.); y_maeda@cc.tuat.ac.jp (Y.M.); tmatsuna@cc.tuat.ac.jp (T.M.)

**Keywords:** chemical mutagenesis, microalgae, biofuels, lipid production, water surface-floating

## Abstract

Water surface-floating microalgae have great potential for biofuel applications due to the ease of the harvesting process, which is one of the most problematic steps in conventional microalgal biofuel production. We have collected promising water surface-floating microalgae and characterized their capacity for biomass and lipid production. In this study, we performed chemical mutagenesis of two water surface-floating microalgae to elevate productivity. Floating microalgal strains AVFF007 and FFG039 (tentatively identified as *Botryosphaerella* sp. and *Chlorococcum* sp., respectively) were exposed to ethyl methane sulfonate (EMS) or 1-methyl-3-nitro-1-nitrosoguanidine (MNNG), and pale green mutants (PMs) were obtained. The most promising FFG039 PM formed robust biofilms on the surface of the culture medium, similar to those formed by wild type strains, and it exhibited 1.7-fold and 1.9-fold higher biomass and lipid productivities than those of the wild type. This study indicates that the chemical mutation strategy improves the lipid productivity of water surface-floating microalgae without inhibiting biofilm formation and floating ability.

## 1. Introduction

Microalgal biofuels have been recognized as a promising alternative to petroleum owing to their high productivity of biomass and lipids compared with that of terrestrial crop plants, no competition with food and feed productions that require extensive arable land, and the advantage of CO_2_ fixation through photosynthesis [1,2,3,4]. Although high energy consumption during the production of microalgal lipids has long hampered the industrial production of microalgal biofuels, researchers have recently explored routes to circumvent this issue [5]. To minimize energy consumption, the methods for harvesting and drying microalgal biomass must be improved; these processes have been estimated to consume more than 69% of the entire energy input for biodiesel production [6]. Several alternative methods such as filtration [7,8,9], gravity sedimentation [10,11], affinity sedimentation [12], flocculation [13], and flotation [14,15] have been proposed to replace centrifugation as the harvesting technique. 

We have previously proposed a novel approach for efficient biomass harvesting in which the water surface-floating microalgal *Botryosphaerella* sp. AVFF007 was employed for lipid production [16]. The strain AVFF007 forms robust biofilms that float on the surface of the culture medium. The floating biofilms can be harvested by adsorption onto poly(ethylene) (PE) films. This approach is promising because of the ease of the harvesting process and lower moisture content of the floating biofilm, which is largely correlated to the amount of energy input required for the drying process. However, lipid productivity of this strain was not higher than that of other microalgae. Because the water surface-floating microalgae found in natural habitats have exhibited a moderate oleaginous phenotype [16], improvement of the biomass and lipid productivities are needed.

Chemical mutagenesis has long been employed to improve the biomass and/or lipid productivities of microalgae such as *Nannochloropsis* sp. [17], *Chlorella vulgaris* [18], *Desmodesmus* sp. [19], and *Chlamydomonas perigranulata* [20]. In particular, it has been demonstrated that the attenuation of the light-harvesting property by mutation could improve the photosynthetic efficiency of microalgae by decreasing the cell-shading effect, allowing an increase in biomass and lipid productivities [21]. However, technical challenges still exist in the mutagenesis of biofilm-forming microalgae because the microalgal biofilms contain an expansive exopolymeric matrix that might decrease the effect of mutagens. In addition, tight cell aggregation in a biofilm is problematic in the screening step following mutagenesis. In a traditional chemical mutagenesis protocol, the target cells are treated with mutagens and subjected to colony formation on agar media. Subsequently, the clones that exhibit the desired phenotypes are screened. Recently, alternative high-throughput screening methods based on flow cytometry were developed [17,22]. However, in any of these methods, the mutated cells need to be adequately dispersed, without which it is difficult to isolate mutated cells with the desired phenotype from the adhesive and surrounding non-mutated cells. 

In this study, we established an effective method to generate chemical mutants of two water surface-floating microalgae strains, *Botryosphaerella* sp. AVFF007 and *Chlorococcum* sp. FFG039. Prior to chemical mutagenesis, the cells were dispersed by sonication. After the sonicated cells were treated with mutagens, the resulting colonies containing less pigment were isolated. Finally, the biomass and lipid productivities of the isolated mutant clones were investigated. On the basis of these results, the potential of the mutated microalgae for oil production is discussed.

## 2. Results

### 2.1. Characterization of Strain FFG039

**Figure 1:**
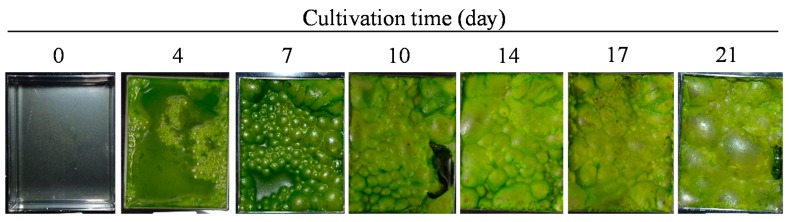
Photographs of *Chlorococcum* sp. FFG039 culture at each cultivation period.

In this study, strain FFG039, in addition to strain AVFF007 [16], was investigated as an alternative oil producer with spontaneous water surface-floating ability. Molecular phylogenetic analysis of the 18S rRNA gene revealed that this strain is closely related to *Chlorococcum aquaticum* UTEX2222. Therefore, we tentatively designated this strain as *Chlorococcum* sp. FFG039, although *Chlorococcum* spp. are not normally biofilm-forming microalgae. *Chlorococcum* sp. FFG039 began to form biofilms at the interface between the culture medium and air after seven days of cultivation, and it covered almost the entire culture medium surface after 10 days (Figure 1). Biofilms were observed to float along with gas bubbles, and were easily harvested from the medium surface by using a PE film, as previously reported [16]. Although the developed biofilms might hamper the light penetration and gas exchange for photosynthesis of the non-floating cells beneath the biofilms, they could recover after the harvesting the biofilms. Subsequently, new biofilms can develop again. The moisture content of the floating biomass was 84.5 ± 0.5%, which was lower than that of the non-floating biomass (90.5 ± 1.2%). This result suggests that the floating ability provides an advantage in biofuel production because a lower energy input is required to dry biomass with a lower moisture content [16]. The comparison of the biomass and lipid productivities of AVFF007 and FFG039 is shown in Table 1. The biomass and lipid productivities of strain AVFF007 were higher than that of FFG039, whereas the lipid content of FFG039 was higher than that of AVFF007. 

### 2.2. Mutagenesis of Water Surface-Floating Microalgae

To improve the lipid productivity of water surface-floating microalgae, AVFF007 and FFG039 were exposed to chemical mutagens ethyl methane sulfonate (EMS) or 1-methyl-3-nitro-1-nitrosoguanidine (MNNG). Prior to mutagen exposure, the cells were sonicated to promote dispersion. The cells of both strains exhibited substantial aggregation but were well-dispersed after 30 s of sonication (Figure S1). Following sonication, the cultures were exposed to varying concentrations of EMS or MNNG, and viability rates were analyzed by a colony formation test. Exposure of FFG039 to 0.25–0.5 M EMS and 0.25–0.5 mM MNNG resulted in viability between 0% and 10% (Figure S2), suggesting that these concentration ranges were appropriate for mutagenesis. 

On the basis of these results, we established chemical mutant libraries of the water surface-floating microalgae (Table S1). EMS or MNNG treatment for AVFF007 generated 33,250 and 63,889 colonies, respectively. The same treatment for FFG039 generated 1688 and 51,970 colonies, respectively. Compared to the wild type colonies, six and 13 colonies in the EMS-treated and MNNG-treated groups of AGFF007, respectively, as well as two and 56 colonies in the EMS-treated MNNG-treated groups of FFG039, respectively, exhibited a pale green color. Since mutagenesis of FFG039 generated pale green mutants (PMs) more efficiently than AVFF007, we further characterized FFG039 PMs.

### 2.3. Characterization of FFG039 PMs

**Figure 2:**
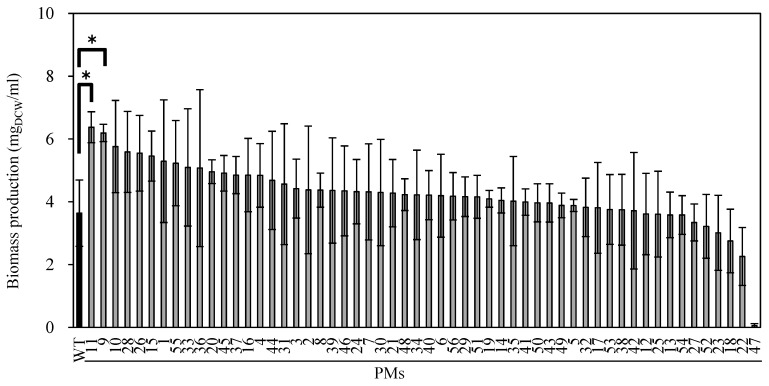
Biomass production of *Chlorococcum* sp. FFG039 wild type (WT) and pale green mutants (PMs) in the 48-well plate. The numbers stand for clone numbers. PMs 1–54 were generated by 1-methyl-3-nitro-1-nitrosoguanidine (MNNG) treatment, and PMs 55–56 were generated by ethyl methane sulfonate (EMS) treatment. Error bars represent standard deviations of three independent experiments.

**Figure 3:**
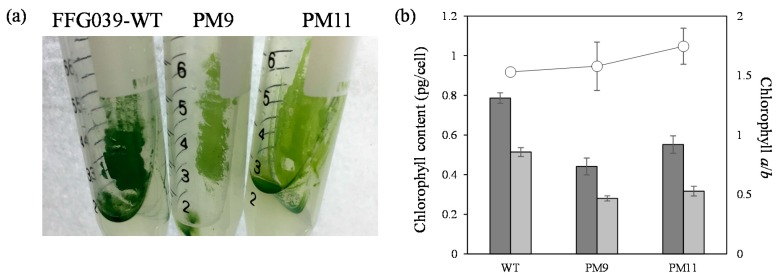
Slant cultures (**a**) and chlorophyll content (**b**) of *Chlorococcum* sp. FFG039 wild type, PM9, and PM11. Error bars represent standard deviations of three independent experiments. The content of chlorophyll *a* (dark gray bars) and *b* (light gray bars) was estimated from the light absorbance at 645 nm and 663 nm, respectively. The ratios of chlorophyll *a*/*b* (open circles) were calculated.

FFG039 PMs were cultured in 48-well microtiter plates containing liquid CSiFF04 medium for 14 days, and their biomass productivity was compared (Figure 2). The biomass productivity of PMs varied from 0.62-fold to 1.75-fold as compared to that of the wild type, with the exception of PM47 (0.02-fold of wild type productivity). Among the 58 PM clones, 46 clones (79%) showed biomass productivity higher than that of the wild type, whereas the difference between the wild type and each mutant was not statistically significant (Student’s *t*-test, *p* > 0.05) except for PM9 and PM11. The PM9 and PM11 exhibited the highest biomass productivity (1.70-fold and 1.75-fold higher than the wild type, respectively). It was confirmed that PM9 and PM11 maintained the water surface-floating ability and that the resulting biofilms could be harvested by a PE film, suggesting that the chemical mutagenesis did not disrupt the advantageous phenotype of this water surface-floating microalga. The amounts of chlorophyll *a* and chlorophyll *b* in PM9 and PM11 were lower than those in the wild type. Therefore, the pale green color of the mutants can be explained by less chlorophyll content (Figure 3). The ratio of chlorophyll *a*/*b* in the wild type, PM9, and PM11 was comparable, and the differences were not statistically significant (Figure 3). The biomass and lipid productivities of FFG039-wild type, PM9, and PM11 were also compared. The lipid content of the wild type and mutants was not significantly different; however, the biomass productivity of the mutants was much higher than that of the wild type (Table 1). Consequently, the lipid productivity of PM9 and PM11 increased by 1.8- and 1.9-fold, respectively, compared with that of the wild type. 

## 3. Discussion

It has long been known that some algal strains (including cyanobacteria and eukaryotic micro- and macroalgae) occasionally form floating clusters (biofilms) on the water surface and play a substantial role in a variety of ecosystems [23,24,25,26]. However, little is known about the potential of floating algae for biotechnological applications. Recently, biofuel production research using floating microalgae was launched, and critical advantages were proposed, such as ease of harvesting [16] and concomitant in situ bioremediation of metals (e.g., Mn, As, Ni, Cr, and Cu) [25].

In our previous study [16], we isolated 168 microalgal strains with water surface-floating ability. Of these, *Botryosphaerella* sp. AVFF007 was selected as one of the most promising candidates for mutagenesis. However, the lipid content of AVFF007 was moderate, and thus this strain may not be the best candidate. In this study, we investigated another water surface-floating microalga, *Chlorococcum* sp. FFG039, as a candidate for mutagenesis, owing to its higher lipid content, compared to that that of AVFF007 (Table 1). Molecular phylogenetic analysis of the 18S rRNA gene sequence revealed that FFG039 is closely related to *Chlorococcum aquaticum* UTEX2222. It was reported that *Chlorococcum aquaticum* UTEX2222 contained a large number of oil bodies, as does FFG039 (Figure S3) [27]. Marine strains belonging to the genus *Chlorococcum* (e.g., *Chlorococcum littorale*) were also studied for the production of biodiesel [28,29], ethanol [30], and hydrogen gas [31]. However, none of these studies have reported the generation of robust biofilms along with gas bubbles and the resulting water surface-floating ability. Therefore, this phenotype is likely uncommon in microalgae belonging to the *Chlorococcum* species and could be a unique characteristic of FFG039. The biofilm of *Botryosphaerella* sp. AVFF007 also captures gas bubbles, which could, in part, contribute to its water surface-floating ability [16], suggesting that these strains might share similar floating mechanisms.

In this study, chemical mutagenesis of water surface-floating microalgae was demonstrated to improve their biomass and lipid productivities. The biofilms on the surface of the culture medium may attenuate light penetration and inhibit photosynthesis of the cells beneath the biofilms. Therefore, it is reasonable to select the mutants with less photosynthetic pigment for better light penetration. This strategy has frequently been employed to improve the productivity of microalgal biomass in large-scale photobioreactors [21], whereas this study, to the best of our knowledge, is the first to apply this strategy to water surface-floating microalgae. The microalgal cells were dispersed by sonication and then exposed to mutagens, following which the PMs were selected. As expected, the selected PMs exhibited higher biomass productivity than the wild type, and their lipid content was comparable to that of the wild type, leading to higher lipid productivity than the wild type. PM11, which is the most promising clone among the PMs, exhibited a biomass productivity of 5.4 ± 0.2 g/(m^2^ day), and a lipid productivity of 1.9 ± 0.1 g/(m^2^ day), which were 1.7-fold and 1.9-fold higher than those of the wild type. These productivities were also higher than those of AVFF039, which had productivities comparable to those of other green microalgae (Table S2) [16]. PM11 retained the biofilm formation and floating phenotypes, suggesting that the chemical mutation did not impair the advantage of the water surface-floating microalgae in biofuel production. 

In this study, we selected gravimetry as a method of quantification of dry biomass and lipids, because a number of studies have employed the same method [17,18,19,32,33,34] and confirmed that the relative standard deviation (RSD) is usually less than 10%. We should note that other methods are also worthwhile for better quantification, such as thin layer chromatography (TLC), in which stained lipid spots are analyzed by imaging tools [35], and gas-chromatography mass spectrometry (GC-MS), in which respective target ion responses are measured [36]. Although the gravimetry data might be still preliminary, this study highlights the promise of the mutant strains for improved biofuel production. In future studies, further investigations will be performed to add more evidence.

## 4. Materials and Methods

### 4.1. Strains and Growth Conditions

Unicellular green microalgae, designated AVFF007 and FFG039, were isolated from freshwater ponds in Kyoto and Nara, Japan, respectively. The two strains were maintained in CSiFF04 medium [16] under continuous illumination of 50 µE/(m^2^ s) at 25 °C with shaking (125 rpm). Cells were subjected to chemical agent mutagenesis followed by the determination of the chlorophyll content. Stationary cultivation for biofilm formation on the surface of the culture medium was performed in 40-mL plastic cases (size: 50 × 63 × 25 mm; AS ONE, Osaka, Japan) arranged in a desiccator. The cells were cultured for approximately one month (initial cell concentration of 1 × 10^5^ cells/mL). CO_2_ gas was provided by adding 0.8 mL ultrapure water to a mixture of 1.25 g sodium hydrogen carbonate and 0.95 g citric acid in a glass vial. The vial was renewed every two days.

### 4.2. Chemical Mutagenesis and Screening

Ethyl methane sulfonate (EMS; Sigma-Aldrich, St. Louis, MO, USA) and 1-methyl-3-nitro-1-nitrosoguanidine (MNNG; Wako Pure Chemical Industries, Ltd., Osaka, Japan) were used as chemical mutagens. Strain FFG039 (1 × 10^6^ cells) in stationary phase was harvested by centrifugation (6000× *g*, 5 min, 4 °C), and the cell pellet was sonicated for 30 s. The percentage of single cells was analyzed by microscopically observing the sonicated cells and calculated by following equation: percentage of single cells = number of single cells observed under microscope/(number of single cells observed under microscope + number of aggregated cells) (Equation (1)). Then, the sonicated cells were suspended in 6 mL of either 0–1 M EMS or 0–1 mM MNNG to induce random mutagenesis. Following incubation for 1 h at room temperature, the same volume of 10% (*w*/*v*) sodium thiosulfate was added to inactivate the mutagens. After the cells were centrifuged and washed three times with CSiFF04 medium, 1 × 10^5^ or 1 × 10^4^ cells were spread on the plates of CSiFF04 medium containing 1% (*w*/*v*) agar to allow each cell to form a colony on the medium agar plates. All colonies appearing at day 14 were counted, and the viability rate was calculated by the following equation: viability rate (%) = average colony number of the cells treated with arbitrary concentrations of mutagens/average colony number of the cells without mutagen treatment (Equation (2)). The colonies that exhibited a pale green color to the naked eye were transferred to 48-well plates (Corning Inc., Corning, NY, USA) containing 1.5 mL of CSiFF04 medium. To screen promising clones among the obtained PMs, the microalgal cells in microtiter plates were filtered using a GA-55 glass fiber filter (retained particle diameter of 0.6 μm; Advantech MFS, Inc., Sierra Court, CA, USA), lyophilized, and weighed.

### 4.3. Quantitative Analysis of Chlorophyll, Biomass, and Lipids

Chlorophyll in microalgal cells was extracted using acetone. The cell pellet (5 × 10^6^ cells) was suspended in 200 µL of acetone and disrupted by sonication (Honda Electronics Co., Ltd., Toyohashi, Aichi, Japan) for 10 min. The supernatant was collected after centrifugation (20,000× *g*, 5 min), and acetone extraction was performed on the cell debris once again. Chlorophyll content was estimated by measuring absorbance of the supernatant at 645 nm (A_645_) and 663 nm (A_663_) using a SH-9000 microplate reader (Corona Electric Co., Ltd., Hitachinaka, Ibaraki, Japan); chlorophyll *a* content (µg/mL) = 12.7 × A_663_ − 2.59 × A_645_; chlorophyll *b* content (µg/mL) = 22.9 × A_645_ − 4.67 × A_663_ [37]. 

After stationary cultivation, biofilms on the culture medium, which are considered floating biomass, were harvested by attachment to PE film several times, as previously reported [16]. Part of the biofilms can tightly adhere to the culture system walls, and those which were not harvested by the attachment of PE film were not considered as floating biomass in this study. The harvested biomass was transferred to an empty tube by using a cell scraper. The harvested biomass was lyophilized, and biomass productivity was calculated on a dry cell weight basis. 

Lipid content in microalgal biomass was determined based on the Folch method [38] with some modification. Approximately 50 mg of dried biomass was ground using a mortar and pestle. Chloroform/methanol (2:1, *v*/*v*, 6 mL in total) was added, and the mixture was agitated by vortexing. Following centrifugation (1000× *g*, 10 min), the supernatant was collected, and the same procedure was repeated for cell debris. Potassium chloride (1.25 mL, 0.1 M) was added to the supernatant and vortexed. The lower layer appearing after centrifugation was transferred to a weighed glass vial, and the solvent was volatilized under an argon atmosphere. 

## 5. Conclusions

Chemical mutagenesis of two strains of water surface-floating microalgae, *Botryosphaerella* sp. AVFF007 and *Chlorococcum* sp. FFG039, was performed. FFG039 mutants with a pale green color (58 clones) were isolated, 46 of which showed higher biomass productivity. The most productive clone, PM11, showed biomass and lipid productivities of 5.4 ± 0.2 g/(m^2^ day) and 1.9 ± 0.1 g/(m^2^ day), respectively, which were 1.7-fold and 1.9-fold higher than those of the wild type. Even after mutation, biofilm formation and water surface-floating abilities were not lost. These results suggest that chemical mutagenesis is a promising way to enhance the potential of water surface-floating microalgae in biofuel production.

## Figures and Tables

**Table 1:** Comparison of the biomass and lipid productivities of water surface-floating microalgae. The biomass was collected after 14 days of cultivation, and the total lipids including neutral lipids and polar lipids were extracted. DCW stands for dry cell weight.

Strain	Biomass Productivity g_DCW_/(m^2^ day)	Lipid Content (%)	Lipid Productivity g_DCW_/(m^2^ day)
*Botryosphaerella* sp. AVFF007	5.2 ± 0.6	24.0 ± 1.1	1.3 ± 0.1
*Chlorococcum* sp. FFG039	3.2 ± 1.0	31.1 ± 2.8	1.0 ± 0.3
*Chlorococcum* sp. FFG039-PM9	5.2 ± 0.4	34.5 ± 2.1	1.8 ± 0.1
*Chlorococcum* sp. FFG039-PM11	5.4 ± 0.2	34.7 ± 0.4	1.9 ± 0.1

## Supplementary Materials

The following are available online at www.mdpi.com/1660-3397/15/6/151/s1. Figure S1. Sonication effect on the cells of *Botryosphaerella* sp. AVFF007 (dark gray) and *Chlorococcum* sp. FFG039 (light gray). AVFF007 and FFG039 were cultured in CSiFF04 medium with vigorous agitation for 14 days. The resulting cell suspension (1 mL, 1 × 10^6^ cells/mL) was subjected to 40 kHz sonication in a bath-type sonicator (Honda Electronics Co., Ltd., Toyohashi, Aichi, Japan). The percentage of single cells was determined by observing the sonicated cells using a microscope and calculating the ratios with Equation (1) (see Materials and Methods). Error bars represent standard deviations of three independent experiments. Figure S2. Viability of *Chlorococcum* sp. FFG039 exposed to EMS (a) and MNNG (b). FFG039 cells (1 mL, 1 × 10^6^ cells/mL, sonicated for 30 s) were exposed to different concentrations of EMS or MNNG for 1 h. To inactivate the mutagens, 10% (*w*/*v*) sodium thiosulfate was added, and the resulting cells were washed with CSiFF04 medium three times. The washed cells were spread onto CSiFF04 agar plates. The colony formation number was counted, and viability rates were calculated by Equation (2) (see Materials and Methods). Error bars represent standard deviations of three independent experiments. Figure S3. Micrographs of the cells of *Chlorococcum* sp. FFG039 wild type and chemical mutants PM9 and PM11 stained with BODIPY505/515. Bright field and fluorescence imaging were performed. Table S1. Chemical mutant libraries of water surface-floating microalgae. Table S2. Comparison of biomass and lipid productivities of microalgae.
