# Supplementary material for: Enhancement of Biomass and Lipid Productivities of Water Surface-Floating Microalgae by Chemical Mutagenesis
Source: Mar Drugs. 2017 May 27;15(6):151. doi: 10.3390/md15060151 (PMC5484101; doi:10.3390/md15060151)
Supplement: Supplementary file 1 [file marinedrugs-15-00151-s001.pdf]

Supplementary Information

Figure S1. Sonication effect of the cells of *Botryosphaerella* sp. AVFF007 (dark gray) and *Chlorococcum* sp. FFG039 (light gray). AVFF007 and FFG039 were cultured in the CSiFF04 medium with vigorous agitation for 14 days. The resulting cells suspension (1 ml, 1 × 10^6 cells/ml) were subjected to 40 kHz sonication in bath-type sonicator (Honda Electronics Co., Ltd, Toyohashi, Aichi, Japan). Percentage of single cells were analyzed by observing the sonicated cells using a microscope, and calculated the ratios by following the equation 1 (see Materials and Method). Error bars represent standard deviations of three independent experiments.

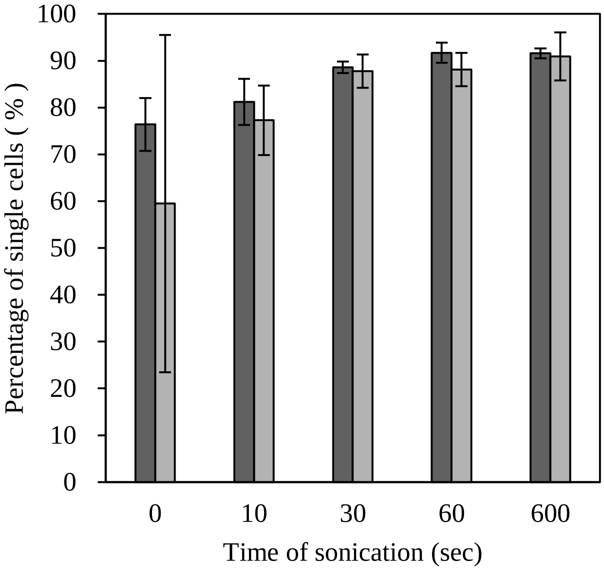

Figure S2. Viability of *Chlorococcum* sp. FFG039 exposed to EMS (a) and MNNG (b). FFG039 cells (1 ml, 1 × 10^6 cells/ml, sonicated for 30 sec) were exposed to different concentration of EMS or MNNG for 1 h. Subsequently, 10% (w/v) sodium thiosulfate were added to inactivate the mutagens, and the resulting cell were washed with CSiFF04 medium 3 times. The washed cells were spread on the CSiFF04 agar plates. The colony formation number were counted, and viability rates were calculated, by following the equation 2 (see Materials and Method). Error bars represent standard deviations of three independent experiments.

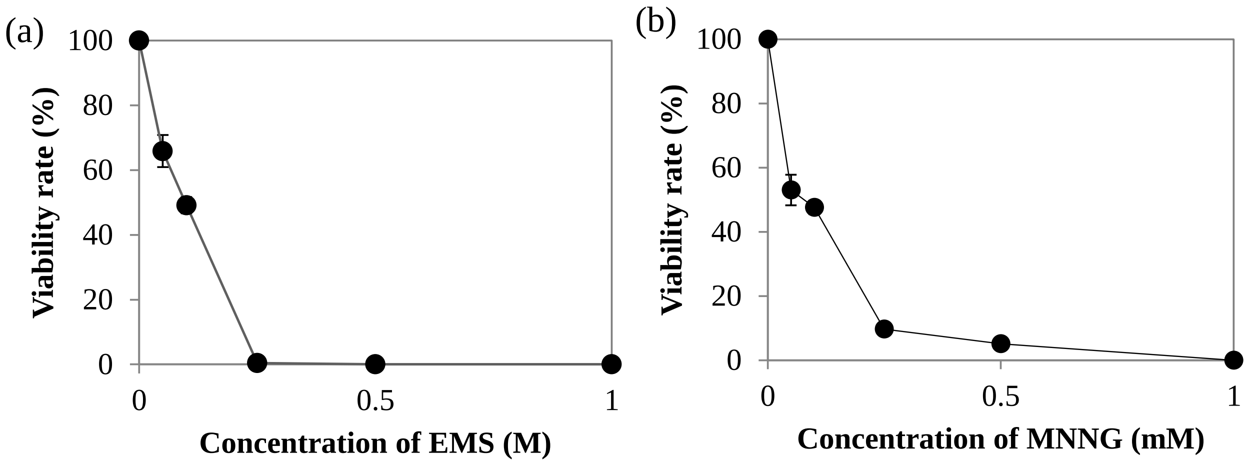

Table S1. Chemical mutant libraries of the water-surface floating microalgae.

Strain	Mutagen	Total colony	Pale-green colony
AVFF007	EMS	33,250	6
	MNNG	63,889	13
FFG039	EMS	1,688	2
	MNNG	51,970	56

Figure S3. Microscopic images of the cells of *Chlorococcum* sp. FFG039 wild type and chemical mutants PM9 and PM11 stained with BODIPY505/515. Bright filed and fluorescence observation was performed.

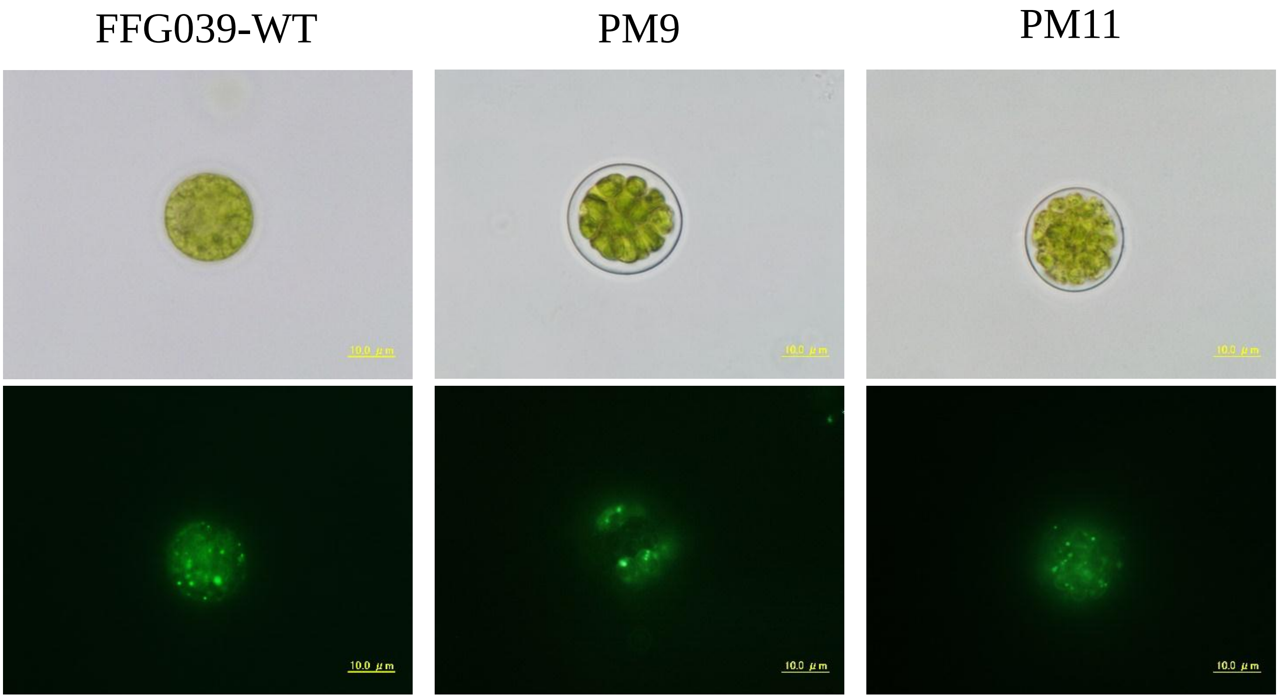

Table S2 Comparison of biomass and lipid productivities of microalgae

Strain	Biomass productivity (g/L/day)	Lipid content (%)	Lipid productivity (mg/L/day)	Reference
Ankistrodesmus falcatus	0.23	30	69	[1]
Chlorella vulgaris	0.24	57	137	
Neochloris oleoabundans	0.29	44	128	
Tetraselmis suecica	0.49	13	64	
Chlamydomonas reinhardtii	0.05	19	10	[2]
Chlorella emersonii	0.29	19	55	
Chlorella salina	0.17	11	19	
Dunaliella salina	0.05	19	10	
Chlorococcum sp. FFG039	0.25 ± 0.08	31.1 ± 2.8	79 ± 24	This study
Chlorococcum sp. FFG039-PM9	0.41 ± 0.03	34.5 ± 2.1	142 ± 8	This study
Chlorococcum sp. FFG039-PM11	0.43 ± 0.02	34.7 ± 0.4	150 ± 8	This study

Reference

1. Griffiths, M. J.; van Hille, R. P.; Harrison, S. T., Lipid productivity, settling potential and fatty acid profile of 11 microalgal species grown under nitrogen replete and limited conditions. *J Appl Phycol* **2012,** 24, (5), 989-1001.
2. Talebi, A. F.; Mohtashami, S. K.; Tabatabaei, M.; Tohidfar, M.; Bagheri, A.; Zeinalabedini, M.; Mirzaei, H. H.; Mirzajanzadeh, M.; Shafaroudi, S. M.; Bakhtiari, S., Fatty acids profiling: a selective criterion for screening microalgae strains for biodiesel production. *Algal Res* **2013,** 2, (3), 258-267.
